# Changes in parent-rated attention-deficit/hyperactivity disorder symptom scores and sleep parameters during individualized multidisciplinary clinical management in children with comorbid obstructive sleep apnea: a prospective cohort study in Vietnam

**DOI:** 10.3389/fsurg.2026.1941040

**Published:** 2026-09-30

**Authors:** Mai Nguyen-Thi-Phuong, Mai Nguyen-Thi-Thanh, Robert Joel Goldberg, Hoa L. Nguyen, Thang Dac Hoang

**Affiliations:** 1 Pediatric Department, Hanoi Medical University, Hanoi, Vietnam; 2 Department of Population and Quantitative Health Sciences, University of Massachusetts Chan Medical School, Worcester, MA, United States; 3 Centre for Toxicology and Radiology Teaching and Research, Vietnam Military Medical University, Hanoi, Vietnam

**Keywords:** attention-deficit/hyperactivity disorder, children, multidisciplinary clinical management, obstructive sleep apnea, respiratory polygraphy, sleep-disordered breathing

## Abstract

**Objective:**

To describe changes in parent-rated attention-deficit/hyperactivity disorder (ADHD) symptom scores, respiratory-polygraphy-derived parameters, and sleep characteristics over 6 months in children with ADHD and comorbid obstructive sleep apnea (OSA) who received individualized multidisciplinary clinical management.

**Methods:**

This single-arm prospective cohort study was conducted at Vietnam National Children's Hospital from November 2022 to October 2024. Among 629 children with ADHD screened using the Pediatric Sleep Questionnaire (PSQ), 293 had at least 8 positive responses and underwent home respiratory polygraphy; 147 had clinical features consistent with pediatric OSA and met the prespecified respiratory-polygraphy criterion of a respiratory event index (REI) ≥ 1 event/hour, and 141 completed follow-up. Parent-rated Vanderbilt ADHD Diagnostic Rating Scale (VADRS) scores were assessed at baseline, 3 months, and 6 months. Respiratory-polygraphy and sleep-diary measures were assessed at baseline and 6 months.

**Results:**

The mean age was 7.8 years, and 85.8% were boys. After 6 months, mean REI decreased from 11.8 to 4.4 events/hour, and the lowest nocturnal peripheral oxygen saturation (SpO₂) increased from 78.9% to 83.4% (both *p* < 0.001). Prespecified respiratory-polygraphy severity categories shifted toward less severe categories (McNemar–Bowker *χ*^2^[6] = 70.80, *p* < 0.001); 73 children (51.8%) improved by at least one category, and 47 children (33.3%; 95% CI, 26.1–41.5%) no longer met the prespecified respiratory-polygraphy criterion for OSA at 6 months. Sleep-onset latency and daytime sleep duration decreased. Nighttime sleep duration increased from 8.4 ± 1.1 to 8.7 ± 0.6 h (mean change, +0.3 h; 95% CI, +0.14 to +0.46; *p* < 0.001), whereas total 24-h sleep duration decreased by 0.5 h (*p* < 0.001). Parent-rated ADHD symptom scores decreased across all VADRS domains.

**Conclusion:**

Respiratory-polygraphy-derived indices, selected sleep characteristics, and parent-rated ADHD symptom scores improved during 6 months of individualized multidisciplinary clinical management. The uncontrolled design and concurrent interventions preclude causal attribution of these changes to OSA-directed treatment.

## 1 Introduction

Attention-deficit/hyperactivity disorder (ADHD) is a common neurodevelopmental disorder in children, characterized by persistent inattention and/or hyperactivity-impulsivity across multiple settings, with substantial adverse effects on academic functioning, behavior, social relationships, and quality of life (1, 2). Children with ADHD frequently experience a broad range of sleep-related problems, including insomnia, sleep terrors, sleepwalking, nightmares, restless legs syndrome, delayed sleep phase disorder, and sleep-disordered breathing (3). These disturbances may exacerbate ADHD symptoms and complicate the distinction between primary ADHD manifestations and the behavioral or cognitive consequences of poor-quality sleep (4, 5).

Obstructive sleep apnea (OSA) is an important form of sleep-disordered breathing in children. It is characterized by recurrent partial or complete upper-airway obstruction during sleep, resulting in impaired ventilation, intermittent hypoxemia, and sleep fragmentation. The prevalence of pediatric OSA is estimated at approximately 1%–5% and is substantially higher among children with adenotonsillar hypertrophy, obesity, allergic rhinitis, asthma, craniofacial abnormalities, or neuromuscular disorders (6–8). Unlike adults, children with OSA do not necessarily present with typical daytime sleepiness; instead, they may exhibit inattention, hyperactivity, impulsivity, irritability, impaired emotional regulation, and poor academic performance (7, 9).

The overlap in clinical manifestations suggests that the relationship between ADHD symptoms and OSA may be complex and potentially bidirectional (4, 7). OSA may produce ADHD-like symptoms or exacerbate the core manifestations of established ADHD (4, 9, 10). Conversely, behavioral difficulties, inappropriate sleep habits, and some medications used to treat ADHD may adversely affect sleep duration and quality (4, 5). Previous studies have suggested that treatment of pediatric OSA may improve not only sleep-related respiratory parameters but also daytime behavior and ADHD-like symptoms. Evidence from randomized and observational studies indicates that adenotonsillectomy may reduce OSA severity and improve behavioral outcomes, although improvements in objective neurocognitive measures have not been consistently demonstrated (10, 11). Recent reviews have also reported potential improvements in ADHD symptoms following adenotonsillectomy and other OSA-directed interventions (4, 9). However, the available evidence remains heterogeneous with respect to study design, follow-up duration, diagnostic criteria for OSA, ADHD assessment tools, and control of comorbid conditions. Moreover, OSA may persist after treatment, particularly in children with obesity or multiple sites of upper-airway obstruction; therefore, objective reassessment is warranted rather than relying solely on symptomatic improvement (6).

In Vietnam, sleep disorders among children with ADHD have received increasing attention. In our previous study of Vietnamese children with ADHD, approximately one quarter of the study sample had OSA with most affected children classified as having moderate-to-severe disease (12). However, available domestic evidence has largely focused on prevalence, clinical characteristics, and associated factors. Prospective data on concurrent changes in respiratory-polygraphy-derived parameters, sleep duration, and parent-rated ADHD symptom scores during comprehensive clinical management remain limited. Therefore, the present study describes changes in parent-rated ADHD symptom scores and sleep parameters in children with ADHD and comorbid OSA during 6 months of individualized multidisciplinary clinical management.

## 2 Methods

### 2.1 Study design

This was a prospective, single-arm, pre–post cohort study conducted at the Department of Psychiatry, Vietnam National Children’s Hospital, from November 2022 to October 2024.

Clinical management decisions were made according to routine practice and were individualized based on ADHD presentation, the prespecified respiratory-polygraphy severity category, and coexisting medical conditions. Participants were assessed at baseline and reassessed at 3 and 6 months after management was initiated.

### 2.2 Participants and participant flow

The study population consisted of children aged 6–12 years who were diagnosed with ADHD and screened for sleep-related breathing problems at the Department of Psychiatry, Vietnam National Children’s Hospital, during the study period.

During the parent study, 629 children with ADHD were screened for suspected OSA using the Pediatric Sleep Questionnaire (PSQ). Of these, 293 had at least 8 positive responses (equivalent to a study score >7) and underwent home respiratory polygraphy. A total of 147 children had clinical features consistent with pediatric OSA and met the prespecified respiratory-polygraphy criterion. Six children were lost to follow-up because they moved abroad, leaving 141 children who completed the required follow-up assessments and were included in the final analysis.

Children were eligible if they were aged 6–12 years at enrollment; were diagnosed with ADHD according to DSM-5 criteria by a child and adolescent psychiatrist (1); had clinical features consistent with pediatric OSA and met the prespecified respiratory-polygraphy criterion of REI ≥1 event/hour (13); had complete baseline parent-rated Vanderbilt ADHD Diagnostic Rating Scale (VADRS), respiratory-polygraphy, and sleep-diary data; and had written informed consent provided by a parent or legal guardian.

Children were excluded if they had a severe neurological or psychiatric disorder that could interfere with ADHD assessment, moderate-to-severe intellectual disability, uncontrolled epilepsy, previous OSA-specific treatment before enrollment, failure to complete the required follow-up outcome assessments, or withdrawal of consent by a parent or legal guardian.

The primary analysis was a complete-case analysis. The six children without the required follow-up assessments were excluded from the analytical sample; no outcome data were imputed.

### 2.3 Study procedures

At baseline, all participants underwent a comprehensive clinical assessment, including medical history taking, child psychiatric evaluation, pediatric examination, and relevant subspecialty assessment when clinically indicated. Baseline procedures included confirmation of ADHD diagnosis and clinical presentation, assessment of parent-rated ADHD symptom severity using the VADRS, screening for suspected OSA using the PSQ, respiratory-polygraphy assessment, completion of a 2-week sleep diary, and evaluation for medical conditions that could contribute to or exacerbate OSA.

Following the baseline assessment, participants underwent individualized multidisciplinary clinical management for ADHD, OSA, and associated medical comorbidities according to recommendations from relevant specialists. Parent-rated VADRS scores were reassessed at 3 months. At 6 months, VADRS, respiratory-polygraphy, and sleep-diary assessments were repeated using the same procedures as at baseline.

### 2.4 Study measures and procedures

#### 2.4.1 Diagnosis and management of ADHD

ADHD was diagnosed according to DSM-5 criteria based on a clinical assessment conducted by a child and adolescent psychiatrist. ADHD was classified into three presentations: predominantly inattentive, predominantly hyperactive-impulsive, and combined.

ADHD symptom severity was assessed using the parent-rated Vanderbilt ADHD Diagnostic Rating Scale (VADRS) (14). Each symptom is rated on a four-point scale: 0 = never, 1 = occasionally, 2 = often, and 3 = very often. A symptom was considered clinically significant when rated as 2 or 3. Subscale scores were calculated by summing the corresponding item scores, with higher scores indicating greater symptom severity (14). For this study, the parent-rated VADRS was administered in Vietnamese. The Vietnamese-language version used in our clinical service was translated from the original English parent form and reviewed by bilingual child psychiatrists and pediatric clinicians to ensure conceptual equivalence and comprehensibility for Vietnamese parents. When necessary, clinicians clarified item wording for caregivers without changing the meaning of the items. The Vietnamese-language VADRS has been used in our clinical service to monitor ADHD symptoms and treatment response. However, because no formally published validation study of the Vietnamese VADRS was available, the scale was used in this study as a standardized parent-reported symptom monitoring instrument rather than as the sole basis for ADHD diagnosis. ADHD diagnosis was established clinically by a child and adolescent psychiatrist according to DSM-5 criteria.

ADHD management was individualized according to symptom severity, clinical presentation, functional impairment, and coexisting conditions. All 141 children received methylphenidate according to routine clinical practice. The treating child psychiatrist determined the initial dose, subsequent dose adjustments, and monitoring of treatment response and adverse effects. Mean daily methylphenidate doses were 18.03 ± 0.53 mg at baseline, 20.22 ± 4.75 mg at 3 months, and 23.72 ± 3.65 mg at 6 months. The study did not assign medication or alter clinical decisions.

#### 2.4.2 Screening for suspected OSA using the pediatric sleep questionnaire

Suspected OSA was screened using the Vietnamese version of the Pediatric Sleep Questionnaire (PSQ), completed by parents or primary caregivers (15, 16). The Vietnamese version had undergone translation and cross-cultural adaptation (15). The PSQ includes 22 items assessing snoring, breathing difficulty during sleep, daytime sleepiness, and behavioral symptoms related to sleep-disordered breathing (16). Each item was scored as follows: “yes” = 1 point, whereas “no” and “do not know” = 0 points. The prespecified screening threshold was a total score >7, meaning at least 8 “yes” responses. Children who met this threshold were referred for objective respiratory assessment (15, 16).

#### 2.4.3 Home respiratory polygraphy and OSA classification

The study definition was informed by the International Classification of Sleep Disorders, Third Edition, Text Revision (ICSD-3-TR) and required clinical features consistent with pediatric OSA together with the prespecified respiratory-polygraphy criterion of REI ≥1 event/hour (13). This operational study definition should not be interpreted as equivalent to a polysomnography-based ICSD-3-TR diagnosis. Respiratory polygraphy was performed with the ApneaLink Plus device at baseline and after 6 months. The device recorded nasal airflow, respiratory effort, heart rate, and peripheral oxygen saturation (SpO₂). Parents or caregivers received direct instruction on device placement and operation before home recording.

A recording was considered technically adequate when it contained at least 4 h of valid data. Every recording was manually reviewed and scored by a sleep specialist at Vietnam National Children's Hospital; artifact and technically invalid signal periods were excluded from the analyzed recording time.

Respiratory events were scored using pediatric criteria consistent with American Academy of Sleep Medicine recommendations (17). An apnea required a ≥ 90% reduction in airflow for at least two breaths. A hypopnea required a ≥ 30% reduction in airflow for at least two breaths with a ≥ 3% oxygen desaturation. Hypopneas associated only with electroencephalographic arousal could not be identified because respiratory polygraphy does not include electroencephalography.

The principal respiratory-polygraphy-derived parameters were the respiratory event index (REI), calculated as the number of scored apneas and hypopneas per hour of valid recording time, and the lowest nocturnal SpO₂ recorded during the monitoring period. Respiratory-polygraphy severity categories were: below the OSA criterion, REI <1 event/hour; mild, 1≤ REI <5; moderate, 5≤ REI <10; and severe, REI ≥10 events/hour (13).

Pediatric studies support the feasibility and screening utility of ApneaLink or home respiratory polygraphy in selected children, but accuracy varies by age and disease severity, and respiratory polygraphy may underestimate polysomnographic apnea–hypopnea index (AHI) (18–20). The American Academy of Sleep Medicine does not recommend home sleep apnea testing as a general substitute for polysomnography in children (21). Accordingly, polysomnography remains the reference standard, and the present results are interpreted as respiratory-polygraphy-derived indices and study-defined severity categories rather than full polysomnographic measurements.

#### 2.4.4 Sleep diary

Children and their parents or legal guardians completed a 2-week sleep diary at baseline and again after 6 months of clinical management. The recorded variables included daytime sleep duration, nighttime sleep duration, bedtime, sleep-onset latency, and total sleep duration over 24 h.

Sleep-onset latency was defined as the interval, in minutes, between going to bed and falling asleep. Total 24-h sleep duration was calculated as the sum of nighttime and daytime sleep. Nighttime sleep duration was calculated separately for each participant at baseline and at the 6-month follow-up by subtracting daytime sleep duration from total 24-h sleep duration. Bedtime was converted to decimal clock time. To preserve chronological ordering when a clock time crossed midnight, post-midnight times were coded by adding 24 h (for example, 00:30 was coded as 24.5) before analysis.

#### 2.4.5 Diagnosis and management of medical comorbidities

All participants underwent clinical evaluation to identify medical conditions that could contribute to or exacerbate OSA. The recorded comorbidities included gastroesophageal reflux disease, asthma, adenotonsillar hypertrophy, allergic rhinitis, and overweight or obesity.

Clinical management was delivered by the relevant specialists according to routine protocols. All 55 children with gastroesophageal reflux disease received medical management; all 27 children with asthma received asthma-control treatment; all 68 children with allergic rhinitis received medical treatment; and all 57 children with overweight or obesity received dietary, physical-activity, and weight-management counseling. Among 79 children with adenotonsillar hypertrophy, 68 underwent surgery: 38 underwent adenotonsillectomy, 17 tonsillectomy alone, and 13 adenoidectomy alone. Surgery was deferred in the remaining 11 children because coexisting asthma and/or allergic rhinitis required medical stabilization. Weight and height were monitored during follow-up as part of routine clinical care. However, serial age- and sex-standardized BMI-for-age z scores were not prespecified or analyzed as a longitudinal study outcome.

All 141 children also received methylphenidate for ADHD. These clinical management exposures were not mutually exclusive because an individual child could receive several interventions concurrently. A complete cross-tabulation of management combinations was not available; therefore, the independent contribution of any single component could not be estimated.

The treating specialists were not involved in outcome assessment or statistical analysis. Subgroup analyses were exploratory and were interpreted as descriptive within-group changes rather than comparisons of treatment effectiveness.

### 2.5 Study variables and outcomes

Baseline characteristics included age, sex, ADHD presentation, baseline study-defined respiratory-polygraphy severity category, and medical comorbidities.

No single primary outcome was prespecified because this was an exploratory follow-up analysis of a fixed cohort, and no *a priori* sample-size calculation was performed. All eligible children with complete required follow-up data were included. Outcomes comprised changes in REI, lowest nocturnal SpO₂, and study-defined respiratory-polygraphy severity category from baseline to 6 months; sleep-diary variables over the same interval; and parent-rated VADRS inattention, hyperactivity–impulsivity, and oppositional-defiant symptom scores at baseline, 3 months, and 6 months. Comorbidity subgroup analyses were exploratory.

### 2.6 Statistical analysis

Data were entered and analyzed using IBM SPSS Statistics, version 26.0 (IBM Corp., Armonk, NY, USA). Continuous variables are presented as means ± standard deviations, while categorical variables are reported as frequencies and percentages.

Changes in continuous outcomes from baseline to 6 months were analyzed using paired-samples *t* tests. Mean changes were reported with 95% confidence intervals, and effect sizes were expressed as Cohen's *dz*. All descriptive statistics, mean changes, confidence intervals, effect sizes, and p-values for the paired continuous outcomes were calculated from the same complete-case paired dataset. Changes in paired study-defined respiratory-polygraphy severity categories from baseline to 6 months were analyzed using the McNemar–Bowker test.

All 141 participants had complete parent-rated VADRS data at baseline, 3 months, and 6 months. Scores were analyzed using one-way repeated-measures analysis of variance. Pairwise comparisons were adjusted using the Bonferroni method, and overall effect sizes were reported as partial *η*^2^. Bonferroni-adjusted pairwise comparisons were performed for baseline versus 3 months, baseline versus 6 months, and 3 versus 6 months; pairwise changes are reported with 95% CIs and Cohen's *dz*. All reported pairwise *p*-values are Bonferroni-adjusted.

Subgroup analyses according to medical comorbidities were exploratory because comorbidity groups were not mutually exclusive. No between-group or interaction tests were performed; subgroup findings were therefore interpreted as descriptive within-group changes.

All statistical tests were two-sided, and a *p*-value <0.05 was considered statistically significant.

### 2.7 Ethical considerations

The study was conducted in accordance with the principles of the Declaration of Helsinki and was approved by the Institutional Review Board of Hanoi Medical University (approval No. 794/GCN-HĐĐĐNCYSH-ĐHYHN; November 16, 2022). Written informed consent was obtained from the parents or legal guardians of all participating children before enrollment.

## 3 Results

### 3.1 Participant flow and baseline characteristics

During the parent study, 629 children with ADHD were screened for suspected OSA using the PSQ. Of these, 293 had at least 8 positive responses and underwent home respiratory polygraphy; 147 had clinical features consistent with pediatric OSA and met the prespecified respiratory-polygraphy criterion. Six children were lost to follow-up because they moved abroad, leaving 141 children who completed the required follow-up assessments and were included in the complete-case analysis.

The mean age of the analytical sample was 7.8 years, and 121 children (85.8%) were boys. The combined ADHD presentation was the most common, observed in 82 children (58.2%), followed by the predominantly inattentive presentation in 43 (30.5%) and the predominantly hyperactive–impulsive presentation in 16 (11.3%).

At baseline, 52 children (36.9%) were in the study-defined mild respiratory-polygraphy category, 41 (29.1%) in the moderate category, and 48 (34.0%) in the severe category. Adenotonsillar hypertrophy was present in 79 children (56.0%), allergic rhinitis in 68 (48.2%), overweight or obesity in 57 (40.4%), gastroesophageal reflux disease in 55 (39.0%), and asthma in 27 (19.1%). Medical comorbidities were not mutually exclusive (Table 1).

**Table 1:** Baseline characteristics of study participants.

Characteristic		*n* = 141	%
Boys		121	85.8
ADHD presentation	Predominantly inattentive presentation	43	30.5
	Predominantly hyperactive–impulsive presentation	16	11.3
	Combined presentation	82	58.2
OSA severity	Mild OSA	52	36.9
	Moderate OSA	41	29.1
	Severe OSA	48	34.0
Medical comorbidities	Gastroesophageal reflux disease	55	39.0
	Asthma	27	19.1
	Adenotonsillar hypertrophy	79	56.0
	Allergic rhinitis	68	48.2
	Overweight or obesity	57	40.4

Medical comorbidities were not mutually exclusive; therefore, individual participants could have more than one comorbid condition.

ADHD, attention-deficit/hyperactivity disorder; OSA, obstructive sleep apnea.

### 3.2 Changes in respiratory-polygraphy-derived indices and study-defined severity categories

After 6 months of individualized multidisciplinary clinical management, mean REI decreased from 11.8 ± 6.5 to 4.4 ± 2.8 events/hour, corresponding to a mean change of −7.4 events/hour (95% CI, −8.3 to −6.5; Cohen's *dz* = −1.31; *p* < 0.001). The lowest nocturnal SpO₂ increased from 78.9 ± 4.1% to 83.4 ± 4.4%, with a mean change of +4.5 percentage points (95% CI, +3.8 to +5.2; Cohen's *dz* =  + 1.06; *p* < 0.001) (Table 2).

**Table 2:** Changes in respiratory polygraphy findings from baseline to the 6-month follow-up.

Clinical subgroup/parameter (mean *±* SD)	Baseline	6-month follow-up	Mean change	95% CI	Cohen's d_z_	*p-*value
Gastroesophageal reflux disease, (n = 55)						
REI *(events/hour)*	12.8 ± 4.6	3.9 ± 2.3	−8.9	−10.0 to −7.8	−2.23	<0.001
Lowest nocturnal SpO₂ *(%)*	78.5 ± 4.3	82.8 ± 4.1	+4.3	+3.2 to +5.4	+1.02	<0.001
Asthma (n = 27)						
REI *(events/hour)*	5.4 ± 3.1	1.5 ± 0.9	−3.9	−5.0 to −2.8	−1.41	<0.001
Lowest nocturnal SpO₂ *(%)*	78.7 ± 9.0	90.7 ± 5.6	+12.0	+8.9 to +15.1	+1.52	<0.001
Adenotonsillar hypertrophy (n = 79)						
REI *(events/hour)*	13.9 ± 8.0	3.3 ± 3.2	−10.6	−12.2 to −9.0	−1.52	<0.001
Lowest nocturnal SpO₂ *(%)*	77.7 ± 5.0	83.5 ± 4.4	+5.8	+4.7 to +6.9	+1.23	<0.001
Allergic rhinitis (n = 68)						
REI *(events/hour)*	13.6 ± 8.8	4.1 ± 3.9	−9.5	−11.3 to −7.7	−1.24	<0.001
Lowest nocturnal SpO₂ *(%)*	78.3 ± 4.0	83.8 ± 4.3	+5.5	+4.5 to +6.5	+1.32	<0.001
Overweight or obesity (n = 57)						
REI *(events/hour)*	7.8 ± 5.3	5.9 ± 3.8	−1.9	−3.2 to −0.6	−0.40	0.004
Lowest nocturnal SpO₂ *(%)*	80.3 ± 6.0	84.3 ± 6.7	+4.0	+2.3 to +5.7	+0.63	<0.001
Overall study population (n = 141)						
REI *(events/hour)*	11.8 ± 6.5	4.4 ± 2.8	−7.4	−8.3 to −6.5	−1.31	<0.001
Lowest nocturnal SpO₂ *(%)*	78.9 ± 4.1	83.4 ± 4.4	+4.5	+3.8 to +5.2	+1.06	<0.001

Data are presented as mean ± standard deviation. Mean change was calculated as the 6-month follow-up value minus the baseline value and is presented with its 95% confidence interval. All within-group changes in Table 2 were analyzed using paired-samples t tests. Effect sizes are reported as Cohen's dz, calculated from the paired within-participant change. Negative changes in REI and positive changes in lowest nocturnal SpO₂ indicate improvement. Comorbidity subgroups were not mutually exclusive; therefore, subgroup analyses were exploratory and were not adjusted for multiple comparisons. No formal between-group comparisons were performed; therefore, differences in change magnitude should not be interpreted as differential treatment effects.

REI, respiratory event index; CI, confidence interval; OSA, obstructive sleep apnea; SD, standard deviation; SpO₂, peripheral oxygen saturation.

Exploratory subgroup analyses are presented in Table 2. Significant within-group changes in REI and lowest nocturnal SpO₂ were observed in all clinical subgroups. Numerically larger REI reductions were observed in the gastroesophageal reflux disease, adenotonsillar hypertrophy, and allergic rhinitis subgroups; however, no formal between-group comparisons were performed. Because the subgroups overlapped and baseline REI values differed, these findings should be interpreted as descriptive within-group changes and not as evidence of differential treatment effects.

Prespecified respiratory-polygraphy severity categories shifted significantly toward less severe categories at 6 months (McNemar–Bowker *χ*^2^[6] = 70.80, *p* < 0.001). Overall, 73 of 141 children (51.8%) improved by at least one category. At 6 months, 47 children (33.3%; 95% CI, 26.1–41.5%) no longer met the prespecified respiratory-polygraphy criterion for OSA (REI <1 event/hour) (Table 3).

**Table 3:** Transition in respiratory-polygraphy severity categories from baseline to the 6-month follow-up.

Baseline severity, *n* (%) (*n* = 141)	Below criterion (REI <1)	Mild OSA	Moderate OSA	Severe OSA	Total
Below criterion (REI <1)	0	0	0	0	0
Mild OSA	15 (28.8)	37 (71.2)	0	0	52 (36.9)
Moderate OSA	12 (29.3)	11 (26.8)	17 (41.5)	1 (2.4)	41 (29.1)
Severe OSA	20 (41.7)	11 (22.9)	4 (8.3)	13 (27.1)	48 (34.0)
Total	47 (33.3)	59 (41.8)	21 (14.9)	14 (9.9)	141

Data are presented as *n* (row percentage). Percentages in the total row were calculated using the total analytical sample (*n* = 141). Changes in respiratory-polygraphy severity categories were analyzed using the McNemar–Bowker test. At 6 months, 47 children no longer met the prespecified respiratory-polygraphy criterion for OSA (REI <1 event/hour). The 95% confidence interval for this proportion was calculated using the Wilson score method.

OSA, obstructive sleep apnea; REI, respiratory event index. Respiratory-polygraphy categories were: below criterion, REI <1; mild, 1≤ REI <5; moderate, 5≤ REI <10; and severe, REI ≥10 events/hour.

### 3.3 Changes in sleep duration and timing

Sleep-diary data showed that daytime sleep duration decreased from 1.7 ± 0.8 to 0.9 ± 0.4 h (mean change, −0.8 h; 95% CI, −0.92 to −0.68; Cohen’s *dz* = −1.15; *p* < 0.001), and sleep-onset latency decreased from 34.7 ± 26.2 to 15.7 ± 10.6 min (mean change, −19.0 min; 95% CI, −22.80 to −15.20; Cohen’s *dz* = −0.83; *p* < 0.001).

Bedtime shifted earlier from 22.3 ± 0.7 to 22.0 ± 0.5 h (mean change, −0.3 h; 95% CI, −0.40 to −0.20; Cohen's *dz* = −0.48; *p* < 0.001). Nighttime sleep duration increased from 8.4 ± 1.1 to 8.7 ± 0.6 h (mean change, +0.3 h; 95% CI, +0.14 to +0.46; Cohen's *dz* =  + 0.31; *p* < 0.001). In contrast, total sleep duration over 24 h decreased from 10.1 ± 1.3 to 9.6 ± 0.7 h (mean change, −0.5 h; 95% CI, −0.69 to −0.31; Cohen's *dz* = −0.44; *p* < 0.001) (Table 4).

**Table 4:** Changes in sleep duration and timing during 6 months of individualized multidisciplinary clinical management.

Sleep duration and timing (*n* = 141)	Baseline	6-month follow-up	Mean change	95% CI	Cohen's dz	*p-*value
Daytime sleep duration *(hours)*	1.7 ± 0.8	0.9 ± 0.4	−0.8	−0.92 to −0.68	−1.15	<0.001
Nighttime sleep duration *(hours)*	8.4 ± 1.1	8.7 ± 0.6	+0.3	+0.14 to +0.46	+0.31	<0.001
Bedtime *(clock time)*	22.3 ± 0.7	22.0 ± 0.5	−0.3	−0.40 to −0.20	−0.48	<0.001
Sleep-onset latency *(minutes)*	34.7 ± 26.2	15.7 ± 10.6	−19.0	−22.80 to −15.20	−0.83	<0.001
Total sleep duration over 24 h *(hours)*	10.1 ± 1.3	9.6 ± 0.7	−0.5	−0.69 to −0.31	−0.44	<0.001

Data are presented as mean ± standard deviation. Mean change was calculated as the 6-month follow-up value minus the baseline value and is presented with its 95% confidence interval. Differences between baseline and follow-up values were analyzed using paired-samples t tests. Effect sizes are reported as Cohen's dz, calculated as the mean within-participant change divided by the standard deviation of the within-participant changes. Negative mean changes indicate reductions in the corresponding sleep parameters. Bedtime was analyzed as decimal clock time; a negative change indicates an earlier bedtime. All tests were two-sided.

Post-midnight bedtime values were coded by adding 24 h (for example, 00:30 was coded as 24.5) before analysis.

### 3.4 Changes in parent-rated ADHD symptom scores assessed using the VADRS

Parent-rated VADRS scores decreased over time in all three domains. Inattention scores decreased from 18.7 ± 4.6 at baseline to 15.9 ± 2.8 at 3 months and 14.4 ± 2.2 at 6 months, with a significant overall time effect [F(2, 280) = 105.61, *p* < 0.001, partial *η*^2^ = 0.43]. Hyperactivity–impulsivity scores decreased from 17.3 ± 4.9 to 14.5 ± 3.0 and 13.3 ± 2.5 [F(2, 280) = 82.22, *p* < 0.001, partial *η*^2^ = 0.37]. Oppositional-defiant symptom scores decreased from 10.2 ± 4.9 to 7.1 ± 3.9 and 5.5 ± 3.5 [F(2, 280) = 89.51, *p* < 0.001, partial *η*^2^ = 0.39] (Table 5, Panel A). Mean daily methylphenidate dose was 18.03 ± 0.53 mg at baseline, 20.22 ± 4.75 mg at 3 months, and 23.72 ± 3.65 mg at 6 months.

**Table 5:** Changes in parent-rated ADHD symptom scores from baseline to the 3- and 6-month follow-ups.

Panel A. Observed scores and omnibus changes over time						
Symptom domain (*n* = 141)	Baseline	3-month follow-up	6-month follow-up	F (2, 280)	Overall p-value	Partial η^2^
Inattention	18.7 ± 4.6	15.9 ± 2.8	14.4 ± 2.2	105.61	<0.001	0.43
Hyperactivity—impulsivity	17.3 ± 4.9	14.5 ± 3.0	13.3 ± 2.5	82.22	<0.001	0.37
Oppositional—defiant	10.2 ± 4.9	7.1 ± 3.9	5.5 ± 3.5	89.51	<0.001	0.39

Panel B. Bonferroni-adjusted pairwise changes between assessment points					
Symptom domain (*n* = 141)	Comparison	Mean change	95% CI	Cohen's *dz*	*p-value*
Inattention	3 months−baseline	−2.80	−3.47 to −2.13	−0.70	<0.001
	6 months−baseline	−4.30	−4.96 to −3.64	−1.08	<0.001
	6 months−3 months	−1.50	−1.93 to −1.07	−0.59	<0.001
Hyperactivity—impulsivity	3 months−baseline	−2.80	−3.51 to −2.09	−0.65	<0.001
	6 months−baseline	−4.00	−4.71 to −3.29	−0.94	<0.001
	6 months−3 months	−1.20	−1.66 to −0.74	−0.43	<0.001
Oppositional—defiant	3 months−baseline	−3.10	−3.85 to −2.35	−0.69	<0.001
	6 months−baseline	−4.70	−5.43 to −3.97	−1.08	<0.001
	6 months−3 months	−1.60	−2.22 to −0.98	−0.43	<0.001

All 141 participants had complete parent-rated VADRS data at baseline, 3 months, and 6 months.

Data are presented as mean ± standard deviation. Overall changes across the three assessments were evaluated using one-way repeated-measures analysis of variance. Mauchly's test did not indicate violation of the sphericity assumption; therefore, uncorrected degrees of freedom are reported as F(2, 280). Pairwise comparisons were performed with Bonferroni adjustment. Mean changes were calculated as the later assessment value minus the earlier assessment value and are presented with 95% confidence intervals. Overall effect sizes are reported as partial η^2^, and pairwise effect sizes as Cohen's dz. Negative mean changes indicate reductions in symptom scores. All pairwise *p*-values shown in Panel B are Bonferroni-adjusted. All tests were two-sided.

ADHD, attention-deficit/hyperactivity disorder; VADRS, vanderbilt ADHD diagnostic rating scale.

Bonferroni-adjusted pairwise comparisons showed reductions from baseline to 3 months, baseline to 6 months, and 3 to 6 months in all three parent-rated domains (all adjusted *p* < 0.001). Baseline-to-6-month mean changes were −4.30 points for inattention (95% CI, −4.96 to −3.64; Cohen's dz = −1.08), −4.00 points for hyperactivity–impulsivity (95% CI, −4.71 to −3.29; Cohen's dz = −0.94), and −4.70 points for oppositional-defiant symptoms (95% CI, −5.43 to −3.97; Cohen's dz = −1.08) (Table 5, Panel B).

## 4 Discussion

### 4.1 Principal findings

This prospective single-arm study found concurrent changes in respiratory-polygraphy-derived indices, selected sleep characteristics, and parent-rated ADHD symptom scores during 6 months of individualized multidisciplinary clinical management in 141 children with ADHD and comorbid OSA. Mean REI decreased, lowest nocturnal SpO₂ increased, and study-defined respiratory-polygraphy severity categories shifted toward less severe categories. More than half of the children improved by at least one category, and one-third no longer met the prespecified respiratory-polygraphy criterion for OSA. Parent-rated VADRS scores decreased by 3 months and showed further reductions between 3 and 6 months. Because participants received several concurrent interventions and there was no comparison group, these changes cannot be attributed specifically to OSA-directed treatment.

### 4.2 Respiratory outcomes and medical comorbidities

Adenotonsillar hypertrophy was the most common medical comorbidity, and children in this subgroup showed marked within-group changes in REI and lowest nocturnal SpO₂. Adenotonsillar hypertrophy is a major cause of upper-airway obstruction in school-aged children (6, 8). Previous studies have shown that adenotonsillectomy can improve respiratory parameters, nocturnal symptoms, and quality of life (11). However, because the subgroups overlapped, baseline severity differed, and no formal between-group comparison was performed, the present subgroup findings do not show that one subgroup improved more than another.

Within-group changes in respiratory-polygraphy-derived parameters were also observed among children with asthma, allergic rhinitis, and gastroesophageal reflux disease. Airway inflammation, mucosal edema, and increased upper-airway resistance are recognized contributors to OSA severity (8, 22). Nevertheless, individual participants could have multiple comorbidities and receive several concurrent interventions. Therefore, the subgroup analyses reflect changes within specific clinical subgroups and should not be interpreted as evidence of the independent effectiveness of any single intervention.

Among children who were overweight or obese, REI and lowest nocturnal SpO₂ also changed significantly within the subgroup. Pediatric OSA associated with obesity is often multifactorial, involving peripharyngeal fat deposition, reduced lung volumes, altered respiratory mechanics, and multilevel upper-airway obstruction (6, 22). These children may remain at risk of residual OSA and may require multimodal management and longer follow-up. Although weight and height were monitored during follow-up, serial age- and sex-standardized BMI-for-age z scores were not analyzed; therefore, the contribution of changes in adiposity to the observed respiratory improvement could not be determined.

### 4.3 Changes in sleep timing and duration

Sleep-onset latency and daytime sleep duration decreased, and bedtime shifted earlier. Nighttime sleep duration increased from 8.4 ± 1.1 to 8.7 ± 0.6 h, corresponding to a modest mean increase of 0.3 h (approximately 18 min; 95% CI, 0.14 to 0.46; Cohen's *dz* = 0.31; *p* < 0.001). In contrast, total sleep duration over 24 h decreased by 0.5 h. This pattern suggests a redistribution of sleep from daytime to nighttime, because the 0.8-hour reduction in daytime sleep was only partially offset by the increase in nighttime sleep. Nevertheless, the clinical significance of the reduction in total sleep remains uncertain. Because the sleep measures were parent-reported, these findings cannot establish improvements in sleep architecture, sleep efficiency, or objective sleep quality.

These changes occurred alongside improvements in respiratory-polygraphy-derived parameters; however, the contribution of OSA-related improvement cannot be separated from the effects of ADHD treatment, comorbidity management, sleep-habit counseling, and other concurrent interventions. Randomized trials in children with OSA have shown that surgical treatment can improve sleep-related symptoms, quality of life, and polysomnographic outcomes, although objective neurocognitive improvements have not been consistently demonstrated (11). In addition, a recent polysomnographic study found that objective sleep abnormalities in children with ADHD and comorbid OSA were more closely related to OSA than to ADHD alone (23). These findings support evaluation of sleep-disordered breathing in children with ADHD who present with sleep complaints.

The observed changes in sleep timing cannot be attributed solely to OSA-directed treatment because participants also received methylphenidate, management of medical comorbidities, and sleep-habit counseling. In addition, sleep variables were derived from parent-reported sleep diaries, while respiratory polygraphy does not assess sleep architecture, sleep efficiency, or cortical arousals. The findings should therefore be interpreted as changes during comprehensive clinical management rather than as definitive evidence of improved overall sleep quality.

### 4.4 Changes in parent-rated ADHD symptom scores and clinical implications

Parent-rated VADRS scores decreased across all three domains at 3 and 6 months, and Bonferroni-adjusted comparisons showed further reductions from 3 to 6 months. This pattern is consistent with previous literature on sleep-disordered breathing and behavior (4, 5, 9, 10). However, all children received methylphenidate, doses increased during follow-up, and many received concurrent OSA-directed and comorbidity management. Caregivers were aware of treatment, which may have introduced reporting or expectancy bias. Therefore, the VADRS findings represent changes in parent-rated symptom scores during multidisciplinary care and do not establish objective improvement in ADHD or an independent effect of OSA-directed treatment.

The finding that one-third of participants no longer met the prespecified respiratory-polygraphy criterion for OSA is clinically relevant. Nevertheless, most children remained within a study-defined OSA category after management. Improvement in parent-rated behavior therefore does not necessarily indicate normalization of sleep-disordered breathing. Children with persistent snoring, mouth breathing, restless sleep, daytime sleepiness, or residual inattention should undergo reassessment, particularly when obesity or multiple coexisting airway disorders are present. Current recommendations similarly emphasize objective follow-up among children at increased risk of residual OSA (6).

Because ADHD itself can adversely affect academic performance, behavior, social relationships, and family functioning, coexisting OSA may further increase the overall burden of disease. The present findings support integrating screening for sleep-disordered breathing into the routine assessment of children with ADHD, particularly those with sleep-related symptoms or upper-airway comorbidities.

### 4.5 Study strengths and limitations

The strengths of this study include its prospective design, relatively large clinical sample, repeated assessments over 6 months, and combined evaluation of parent-rated ADHD symptom scores, sleep timing, and respiratory-polygraphy-derived parameters. The study also reflects real-world individualized multidisciplinary clinical management in which ADHD, OSA, and relevant medical comorbidities were addressed concurrently.

Several limitations should be considered. First, the single-arm design without a comparison group precludes causal inference. Second, participants received multiple concurrent interventions; only counts for individual components were available, not a complete cross-tabulation of combinations, so the independent contribution of each component could not be estimated. Methylphenidate doses also increased during follow-up. Third, the Vietnamese VADRS has not undergone a formally published validation study; it was parent-rated by caregivers who were aware of treatment, creating possible reporting or expectancy bias, and no teacher-rated or objective neuropsychological outcome was available. Fourth, serial age- and sex-standardized BMI-for-age z scores were not included as a longitudinal outcome, limiting assessment of the contribution of changes in adiposity to respiratory outcomes. Fifth, home respiratory polygraphy does not measure true sleep time, sleep stages, sleep efficiency, or cortical arousals and may underestimate polysomnographic AHI, particularly in mild-to-moderate disease. Although recordings were manually reviewed by a sleep specialist, polysomnography remains the reference standard for pediatric OSA diagnosis. Finally, no single primary outcome or *a priori* sample-size calculation was prespecified, and 6 months of follow-up was insufficient to assess durability, recurrence, or long-term academic and social outcomes.

## 5 Conclusions and recommendations

In this single-arm prospective cohort, respiratory-polygraphy-derived indices, selected sleep characteristics, and parent-rated ADHD symptom scores improved during 6 months of individualized multidisciplinary clinical management. More than half of the children improved by at least one prespecified respiratory-polygraphy severity category, and one-third no longer met the prespecified respiratory-polygraphy criterion for OSA at follow-up. The uncontrolled design and concurrent interventions preclude causal attribution of these changes to OSA-directed treatment.

The findings support screening for OSA and related medical comorbidities in children with ADHD, particularly those with sleep-related symptoms or upper-airway risk factors. Controlled studies with validated multi-informant ADHD outcomes, polysomnography, serial anthropometry, and longer follow-up are needed to distinguish the contributions of ADHD treatment, OSA-directed treatment, comorbidity management, and weight control.

## Data Availability

The raw data supporting the conclusions of this article will be made available by the authors, without undue reservation.
